# Characteristics of telogen effluvium in COVID-19 in western Iran (2020)^☆^^☆☆^

**DOI:** 10.1016/j.abd.2021.05.006

**Published:** 2021-09-15

**Authors:** Khaled Babaei, Hossein Kavoussi, Mansour Rezaei, Reza Kavoussi

**Affiliations:** a Dermatology Department, Hajdaie Dermatology Clinic, School of Medicine, Kermanshah University of Medical Sciences (KUMS), Kermanshah, Iran; b Health School, Family Health Research Center of Kermanshah University of Medical Sciences (KUMS), Kermanshah, Iran; c Kermanshah University of Medical Sciences (KUMS), Kermanshah, Iran

**Keywords:** Alopecia, COVID-19, Vitamin D deficiency

## Abstract

**Background:**

Although COVID-19 pandemic significantly induces mortality, many of the patients who recovered present other medical problems such as alopecias. Telogen effluvium is a common alopecia that is usually related to previous events such as acute febrile diseases, including COVID-19.

**Objective:**

To evaluate the characteristics of telogen effluvium in COVID-19.

**Method:**

This cross-sectional study was carried out on 526 patients with documented telogen effluvium that recovered from COVID-19. Demographic data, concurrent alopecia, associated diseases, and COVID-19 severity were recorded. Data were analyzed by appropriate statistical methods.

**Results:**

The mean age of the 526 patients (410 females, 116 males) was 30.97 ± 9.592 years, with 7.65 ± 1.739 weeks of mean time of alopecia onset. Vitamin D deficiency (24.3%), androgenetic alopecia (78.2%), and grade III COVID-19 severity were the most common findings. Alopecia onset was significantly earlier in the younger age group, females, in hypothyroidism, and more severe coronavirus infection. Higher grade coronavirus infection was significantly seen in males, higher ages, earlier onset, and androgenic alopecia.

**Study limitations:**

Performing a single-center study and considering limited variables.

**Conclusion:**

Although Coronavirus 2 infection can be an important factor in telogen effluvium induction, other factors such as associated diseases, drug intake and emotional stress may also be involved. In the cases of early onset of alopecia, concomitant diseases such as hypothyroidism and severe coronavirus infection can occur, thus, the presence of various factors in telogen effluvium induction should be considered.

## Introduction

In late 2019, a coronavirus, i.e., Severe Acute Respiratory Syndrome Coronavirus 2 (SARS-CoV-2), caused Coronavirus Disease-2019 (COVID-19). This disease created public health crises worldwide, causing an ongoing pandemic in most countries with 3%–4% mortality rate.^1^, ^2^

Although COVID-19 is usually associated with mild to severe respiratory diseases in the acute phase, some patients who recover from COVID-19 complain of dermatologic signs and symptoms such as Hair Loss (HL) several weeks after recovery from the disease.^3^, ^4^, ^5^, ^6^

Telogen Effluvium (TE) is a common HL form in which many factors are involved. Acute fever illnesses such as viral infections, including COVID-19, are occasionally involved in the pathogenesis of TE.^6^, ^7^, ^8^, ^9^, ^10^, ^11^

In COVID-19 disease, in addition to the acute febrile disease, other factors such as emotional stress, intake of some drugs, hospitalization, associated diseases, nutritional problems, weight loss, and other factors may be involved in the induced TE.

Due to the pandemic outbreak of COVID-19 and its correlation with TE, the characteristics of TE in the patients who presented to the study’s dermatology clinic with improved COVID-19 were evaluated.

## Method

### Study population

This cross-sectional study was done on 526 patients with documented TE, who recovered from COVID-19, in Hajdaie dermatology clinic in Kermanshah, Iran over 12 months in 2020.

The patients with clinically documented TE after improvement of COVID-19 were enrolled in the present study, but the patients with psychological problems, severe systemic diseases, recent consumption of multiple drugs other than used in COVID-19, postpartum patients, and patients recovering from COVID-19 for more than 6 months were excluded from the study.

After taking written informed consent, demographic data, Duration of Hair Loss Onset After Improvement (DHLOAI), the severity of COVID-19, other concurrent types of alopecia, and laboratory assessment were recorded in the questionnaire.

The ethical committee of Kermanshah University of Medical Sciences approved the present study (IR.KUMS.REC.1398.674). The information of all patients was kept confidential.

### COVID-19 documentation and severity classification

The COVID-19 patients diagnosed by PCR assessment or patients who had respiratory symptoms and close contact with COVID-19 patients were enrolled. COVID-19 disease was classified according to the severity of signs and symptoms as Grade I: patients who had mild to moderate signs or symptoms consistent with an influenza-like disease, Grade II: patients who had moderate to severe signs or symptoms consistent with an influenza-like disease without hospitalization, Grade III: patients who had moderate to severe signs or symptoms consistent with an influenza-like disease and hospitalization in an ordinary ward, Grade IV: patients who had severe signs or symptoms consistent with an influenza-like disease and hospitalization in an intensive care unit (ICU) ward, without respiratory intubation, and Grade V: patients hospitalized in an ICU ward with respiratory intubation and connection to a ventilator.

### Data analysis

SPSS 16.0 software was used for data analysis. The frequency tables (frequency and percentage) were used to summarize the qualitative data. Mean and standard deviation were used to summarize the normally distributed quantitative variables, and median and range (maximum-minimum) were used for non-normally distributed quantitative variables.

Then, cross-tabulation, two-dimensional bar charts, and Pearson’s chi-square test were used to determine the relationship between qualitative variables.

Kruskal-Wallis test was used to compare the median ranks of non-normally distributed quantitative variables between the categories of multinomial qualitative variables, p < 0.05 was set as the significance level.

## Results

In this study, 526 patients, including 410 (77.9%) females and 116 (22.1%) males, were recruited. The age range of the participants was 8–62 years, with a mean age of 30.97 ± 9.592 years and 7.65 ± 1.739 weeks of Mean Duration of HL Onset After Improvement (MDHLOAI) of COVID-19 (Table 1).

**Table 1:** Demographic and other characteristics of patients.

Variables		Results
Mean age of patients (years)		30.97 ± 9.592
Mean duration of disease onset (week)		7.65 ± 1.739
Sex n (%)	Female	410 (77.9%)
	Male	116 (22.1%)
Associated diseases n (%)	Yes	181 (34.4%)
	No	345 (65.6%)
Type of associated diseases or laboratory findings n (%)	Vitamin D deficiency	44 (24.3%)
	Elevated ESR	38 (21.0%)
	Hypothyroidism	38 (21.0%)
	Diabetes	36 (19.9%)
	Iron deficiency anemia	25 (13.8%)
Smoking n (%)	Yes	57 (10.8%)
	No	469 (89.2%)
Alcohol consumption n (%)	Yes	43 (8.2%)
	No	483 (91.8%)
Concurrent alopecia n (%)	Yes	147 (27.9%)
	No	379 (72.1%)
Type of concurrent alopecia n (%)	Androgenic alopecia	115 (78.2%)
	Alopecia areata	28 (19.0%)
	Cicatricial alopecia	4 (2.7%)
Grading of COVID-19 n (%)	Grade I	100 (19.0%)
	Grade II	150 (28.5%)
	Grade III	193 (36.7%)
	Grade IV	83 (15.8%)
	Grade V	0 (0.0%)
Total number of patients		**526 (100.0%)**

Further, 100 (19%), 150 (28.5%), 193 (36.7%), 83 (15.8%), and 0 (0.0%) patients had Grade I to V COVID-19, respectively (Table 1).

Other concurrent types of alopecia-associated diseases, abnormal laboratory findings, and other characteristics of patients are presented in Table 1.

Higher grade COVID-19 was significantly seen in the male patients, patients with shorter DHLOAI, higher ages, and patients with AGA (P < 0.05) (Table 2).

**Table 2:** Association between COVID-19 severity and mean duration of hair loss onset after improvement with some variables.

Variables	Grading of COVID-19				P value	MDHLOAI	P value
	I	II	III	IV			
Sex:							
Female	83(20.2%)	119(29.9%)	156(38.0%)	52(12.7%)	0.03	252.3	0.001
Male	17(14.7%)	31(26.6%)	37(31.9%)	31(26.7%)		303.2^a^	
ADLF:							
Vitamin D deficiency	6(13.6%)	11(25.0%)	21(47.7%)	6(13.6%)	0.250	15.89 ± 3.384	0.000
Hypothyroidism	7(18.4%)	8(21.1%)	16(42.1%)	7(18.4%)		13.79 ± 2.361	
Elevated ESR	6(15.8%)	6(15.8%)	16(42.1%)	10(26.3%)		15.26 ± 2.777	
Diabetes	3(8.3%)	9(25.0%)	16(44.4%)	8(22.2%)		15.81 ± 2.703	
Iron deficiency anemia	4(16.0%)	6(24.0%)	10(40.0%)	5(20.0%)		15.52 ± 3.137^b^	
Concurrent type of alopecia:							
Androgenic alopecia	23(20%)	33(28.7%)	39(33.9%)	20(17.4%)	0.038	15.90 ± 3.115	0.568
Alopecia areata	2(7.1%)	10(35.7%)	11 (39.3%)	5 (17.9%)		15.71 ± 3.196	
Cicatricial alopecia	1(25.0%)	2(50.0%)	1(25.0%)	0(0.0%)		13.75 ± 3.304^b^	
Smoking	7(12.3%)	18(31.6%)	19(33.3%)	13(22.8%)	0.269	259.1 vs 264.0^a^	0.816
Addiction	3(7.0%)	12(27.9%)	17(39.5%)	11(25.6%)	0.089	264.9 vs 248.2^a^	0.489
DHLOAI (week) (Mean ± SD)	8.89 ± 2.039	7.98 ± 1.693	7.12 ± 1.315	6.81 ± 1.273	0.000		
MHL (week)	9.0(3.0)	8.0(2)	7.0(2)	7.0(2)	0.000		
Median (IQR)							

MDHLOAI, Mean duration of hair loss onset after improvement; DHLOAI, Duration of hair loss onset after improvement; MHL, Median of hair loss; IQR, Inter Quartile Range; ADLF, Associated disease or Laboratory findings.

a Mean Rank.

b Mean ± SD.

The DHLOAI was significantly shorter in the younger patients, female patients, and patients with hypothyroidism and a higher grade of COVID-19 (P < 0.05) (Table 2).

Correlation between severity of COVID-19 and other variabilities, also DHLOAI and other variabilities are presented in Table 2.

The median and quartile range for the onset of hair loss according to the severity of COVID-19 are shown in Table 2.

The ordinal logistic regression model for grading COVID-19 and its associated variables are shown in Table 3.

**Table 3:** Ordinal logistic regression model for grading of COVID-19 and its associated variables.

Variables	Coefficient	S.E	P-value	95% CI
COVID-19 Grade I	2.749	0.825	0.001	(1.13, 4.36)
COVID-19 Grade II	4.778	0.834	0.000	(3.14, 6.41)
COVID-19 Grade III	7.542	0.885	0.000	(5.8, 9.27)
Sex	0.904	0.216	0.000	(0.48, 1.32)
Age	0.140	0.012	0.000	(0.11, 0.16)
Concurrent types of alopecia	0.508	0.120	0.000	(0.27, 0.74)
DHLOAI	-1.819	0.221	0.000	(-2.25, -1.38)

DHLOAI, Duration of hair loss onset after improvement; S.E, Std. Error; CI, Confidence Interval.

## Discussion

Although COVID-19 induces TE similar to other acute febrile diseases, the present study showed that DHLOAI was significantly shorter in the younger patients, females, and hypothyroidism. Moreover, severe COVID-19 was significantly more prevalent in males, higher ages, AGA, and early onset of HL.

Most of the patients were female and usually in the third and fourth decades of life, non-smoker, non-alcoholism, and non-addicted, mostly in Grade III of severity, and AGA was the most common concurrent HL, with 7.65 ± 1.739 weeks of MDHLOAI of COVID-19.

In the present study, 77.9% of patients were female, who presented earlier and mostly had a lower grade COVID-19 than the males. The authors think that women present earlier to physicians because of their sensitivity to beauty^12^ and long hair, which makes hair loss more obvious. On the other hand, the high prevalence of some disorders such as emotional stress,^6^ thyroid abnormality^7^, ^8^ and anemia in the females make them prone to earlier beginning of HL due to TE. The mean age of the present study’s patients was 31 years. However, a study reported three senile females with TE, which is related to SARS-CoV-2 infection.^6^ Further, Moreno-Arrones et al.^12^ found the mean age of 47.4 years for the patients that presented with TE after COVID-19 involvement. Although in the global outbreak of COVID-19 all age groups, especially the elderly, are affected, the disease may be more prevalent in some age groups in each geographical area. It seems that in the studied region, the patients with COVID-19 are younger and are more sensitive to HL, so the mean age of patients is lower in the studied region than in other areas.

Evaluation of the study’s patients revealed vitamin D deficiency (24.3%) and hypothyroidism (21%) were the most common findings. Both mentioned disorders are the main cause of HL and TE^6^, ^7^ therefore, they can exacerbate or make HL chronic in association with COVID-19 infection.

The present findings showed hypothyroidism resulted in the early onset of HL after improvement of COVID-19 infection. Therefore, hypothyroidism is a very important factor that accelerates the progression of hair follicles from anagen to telogen phase.

In a study, the MDHLOAI of COVID-19 was 58.6 days,^4^ which was nearly similar to the onset of HL in the patients. The study’s findings also showed patients with severe COVID-19 infection and female and young patients had a significantly earlier referrals. This might be related to the multiplicity of associated factors such as emotional stress, intake of multiple drugs, high fever, and concurrent systemic disorders in severe COVID-19 in the studied patients.

More than 50% of the patients in this present study were Grade III and IV, as the severe disease, that presented with early HL onset. Severe COVID-19 infection may result in high production of proinflammatory cytokines and a proinflammatory state. Furthermore, in this situation, the coagulation cascade is activated and lowers the circulation of anticoagulant agents. These aspects may be associated with microthrombi and occlusion in micro-vessels of the hair follicle. Additionally, the multiplicity of associated risk factors in severe COVID-19 infection lead to the early onset of HL in TE.^13^

Most of the patients were non-smoker, non-alcoholic, and non-addicted, therefore, they had a minor impact on HL. But these variables may lead to HL through secondary effects such as malnutrition, low hygiene, and systemic disorders.

Wambier et al.^14^ showed the majority of hospitalized or severe COVID-19 patients had AGA. AGA was also the most common type of concurrent alopecia in the patients. The importance of the coexistence of TE and AGA is its poor prognosis, which shows the low possibility of a good improvement of hair density.^15^ On the other hand, the high frequency of coexistence of TE and AGA, which was seen in the patients, is associated with more emotional stress, which can consequently exacerbate HL.

In the study’s findings, which are consistent with other studies, higher age and male gender is usually associated with severe involvement of the COVID-19 infection,^2^, ^3^ so it is associated with hospitalization and many secondary complications of viral infection as other risk factors to induction TE. These reasons justify the possibility of more TE and early onset of HL in men and severe cases of COVID-19 infection.

## Conclusion

Although COVID‐19 infection can induce TE like other acute viral infections, many factors such as other associated disorders, multiple drug intake, emotional stress, hospitalization, nutritional problem, and some secondary complications of viral infections as other associated risk factors may be involved in TE triggering. In the cases of early onset of hair loss, the possibility of concomitant disorder such as hypothyroidism and severe coronavirus infection, thus the presence of various factors in telogen effluvium induction should be considered.

## Financial support

None declared.

## Authors’ contributions

Khaled Babaei: Study conception and planning; critical literature review.

Hossein Kavoussi: Approval of the final version of the manuscript; effective participation in research orientation; study conception and planning; manuscript critical review.

Mansour Rezaei: Data collection, analysis and interpretation; satistical analysis.

Reza Kavoussi: Intellectual participation in propaedeutic and/or therapeutic management of studied cases; preparation and writing of the manuscript.

## Conflicts of interest

None declared.
